# Genetic subtypes, allelic effects, and convergent neurodevelopmental mechanisms

**DOI:** 10.1186/s13073-021-00913-y

**Published:** 2021-06-07

**Authors:** Maitreya Das, Santhosh Girirajan

**Affiliations:** Department of Biochemistry and Molecular Biology and of Anthropology, Pennsylvania State University, 205A Life Sciences Building, University Park, PA 16802 USA

**Keywords:** Neurodevelopmental disorders, Autism, Molecular subtypes, Alleles, Stress granules

## Abstract

High-throughput sequencing of large affected cohorts have helped uncover a plethora of risk genes for complex neurodevelopmental disorders. However, untangling complex disease etiology also involves understanding the functional consequences of these mutations in order to connect risk variants to resulting phenotypes. Here, we highlight the efforts of Mannucci and colleagues to define a novel molecular subtype of neurodevelopmental disorder associated with mutations in *DHX30* and characterize location-specific mutational effects in cell culture and zebrafish models.

Advances in genomic technologies and assessment of large cohorts of individuals have implicated multiple genes towards neurodevelopmental disorders. Recently, collaborative efforts by investigators connected through web-based tools such as GeneMatcher (https://genematcher.org) have aggregated sizable numbers of cases with mutations in the same gene, allowing for characterization of phenotypic outcomes along with allelic and functional effects. Using this strategy, several genes have been reported, with each gene causing a subtype of neurodevelopmental disorder. Writing in *Genome Medicine*, Mannucci and colleagues describe a rare subtype of neurodevelopmental disorder caused by mutations in the RNA helicase encoding *DHX30* and characterize location-specific functional effects of different mutations in this gene using molecular assays and behavioral studies in zebrafish [1]. *DHX30* belongs to the Super Family 2 (SF2) of RNA helicases with integral roles in mRNA regulation, transport, storage, and decay—all of which contribute to neuronal differentiation, development, and maturation. DHX30, along with other SF2 members such as DDX3X, DDX6, and DDX59, have been strongly implicated in neurodevelopmental disorders [1]. Based on the concepts in this study, we highlight general themes uncovered when dissecting genetic heterogeneity as well as overlapping and converging mechanisms of genes contributing to neurodevelopmental disorders.

## Allelic heterogeneity: another layer of complexity

While many studies of complex diseases focus on identifying recurrently mutated genes within specifically ascertained disease cohorts, such as autism and schizophrenia, aggregate analysis of individuals has also uncovered a range of genes exhibiting allelic mutations with varying phenotypic effects [2]. For example, Mannucci and colleagues found that individuals carrying missense variants within the helical core motif (HCM), a functional domain within DHX30, tend to manifest severe developmental features, including complete absence of speech, microcephaly, hypotonia, abnormal gait, and sleep disturbance. In contrast, individuals with mutations outside the HCM region displayed significantly less severe features. The authors further matched phenotypic profiles with the location of mutations in *DHX30* and identified more functionally relevant patterns of correlation. For instance, loss-of-function variants, such as frameshift and nonsense mutations or whole gene deletions, only resulted in milder phenotypes, suggesting distinct effects on protein function due to missense variants in specific locations. Although in vitro experiments did not paint a clear picture, overall trends suggested that missense mutations led to a significant loss of ATPase and helicase activity, reduction in global translation, and formation of stress granules [1].

These observations are reminiscent of location-specific mutational (or allelic) effects reported for other genes that have been strongly associated with neurodevelopmental disorders. For example, the position of mutations along *NRXN1* was shown to correlate with specific clinical outcomes [3]. Studies on intragenic deletions within *NRXN1* found that individuals with exonic deletions towards the C-terminal end of the gene were more likely to manifest macrocephaly (head sizes > 90th percentile) and seizures (> 85%) compared to those with N-terminal deletions [3]. In another study, Beunders and colleagues reported on 24 individuals with exonic deletions of *AUTS2* and manifesting variable set of features, including intellectual disability, autism, microcephaly, short stature, and facial dysmorphism [4]. Interestingly, the authors observed that individuals with deletions encompassing the 3’ end of *AUTS2*, encoding the C-terminal isoform of AUTS2 and expressed in the brain, tended to manifest more syndromic features such as microcephaly and facial dysmorphism, which were further recapitulated in zebrafish models for the C-terminal isoform [4]. These cases exemplify that many neurodevelopmental genes are likely to exhibit variable functional and phenotypic effects conferred by location of mutations, although we have yet to explore the range of these effects in most genes.

## Molecular subtypes and functional convergence

**Fig. 1:**
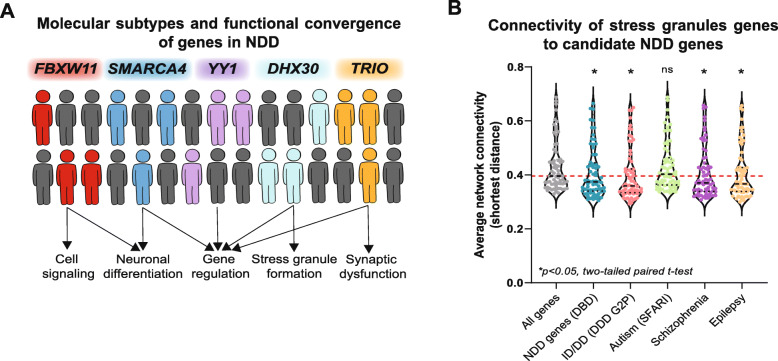
**A** Schematic shows that while variants in multiple candidate genes each contribute a small effect size towards cases of neurodevelopmental disorders, they may each affect overlapping molecular functions, providing a shared etiology for distinct subtypes of developmental disorders. **B** Violin plots show the average connectivity of 68 cytoplasmic stress granule genes, annotated from the Gene Ontology database (GO:0010494), with candidate neurodevelopmental genes from disorder-specific databases (DBD: https://dbd.geisingeradmi.org/; DDDG2P: https://www.ebi.ac.uk/gene2phenotype/; SFARI: https://gene.sfari.org/; Schizophrenia [6]; Epilepsy [7], in the context of a human brain-specific interaction network. Average connectivity was calculated as the shortest distance between two genes in the network, which was previously constructed using a Bayesian classifier trained on brain co-expression datasets [8]. * indicates gene categories with significantly less connectivity than the average connectivity of stress granule genes with all genes in the genome (p < 0.05, two-tailed paired t tests)

Recent studies have further suggested that extensive genetic heterogeneity, where multiple distinct genes contribute to an underlying phenotype, is observed when populations are ascertained for broader neurodevelopmental manifestations. For example, a study of approximately 35,000 individuals with autism identified de novo gene disruptive mutations in more than 100 genes. Only a small subset of genes has been shown to carry mutations in two or more individuals; each of these genes defines a molecular subtype for the disorder and accounts for less than 0.5% of total cases [5]. In fact, the combined contribution of the top 26 genes implicated in neurodevelopmental disorders, including *CHD8*, *DYRK1A*, and *DDX3X*, was estimated to be only about 0.04% [2]. However, further studies on the discovered genes suggest patterns of functional convergence across these seemingly distinct molecular subtypes (Fig. 1A). For example, mutations in the SWI/SNF family of genes, including *ADNP*, *SMARCA4*, and *ARID1B*, affect chromatin remodeling and maturation of post-mitotic neurons [9].

Mannucci and colleagues validated the role of *DHX30* and its protein variants towards formation of stress granules [1]. Stress granules are an aggregation of multiple ribonucleoprotein complexes that pause translation in response to cellular stress [10]. In their study, transfected cells expressing HCM variants showed localization of altered DHX30 to stress granules, resulting in loss of global cellular translation, while unaltered cells showed diffuse expression of wild-type DHX30 throughout the cytoplasm. The authors further used cell culture and zebrafish models to demonstrate that endogenous DHX30 leads to stress granule aggregation under heat stress conditions, while *DHX30* deletion contributes to a reduction in the number of stress granules in these models. In addition, behavioral assessment of *DHX30* knockouts in zebrafish showed altered sleep-wake behavior, which is consistent with patterns of sleep disturbance observed in several individuals carrying *DHX30* variants [1].

Interestingly, stress granule formation has been associated with maintenance of synaptic plasticity through regulation of synaptic activity-dependent local translation and has been linked to genes involved in neurodevelopmental disorders, such as *FMR1* and *DDX3X* [10]. In fact, genes annotated for stress granule-related functions in the Gene Ontology database showed strong connectivity to candidate neurodevelopmental genes, including genes associated with intellectual disability, schizophrenia, and epilepsy, in the context of a brain-specific interaction network (Fig. 1B). These overlaps observed among molecular subtypes uncover novel functional links between different genes and aid in our understanding of complex clinical outcomes [6, 7].

## Solving the genetic heterogeneity puzzle, one gene at a time

Integrative efforts from researchers all over the world aided by sequencing-based genetic screening and social media tools such as GeneMatcher have been successful in collating rare disruptive mutations for individual genes from diverse cohorts. These efforts have mustered large sample sizes and detailed clinical characterization for rare subtypes of disorders, which otherwise would have resulted in single case reports with limited diagnostic utility. More than 400 studies have used GeneMatcher, indicating the extensive impact of this collaborative concept.

In conclusion, we amplify the importance of rare variant studies and highlight how characterization of allelic variants reveals additional functional complexity of candidate genes. Studies focused on genetic subtypes provide a unique opportunity to dissect each variant by type and location in order to pinpoint causal links between variant-specific functions and phenotypic outcomes. Also, a collective look at these studies will help identify functionally convergent patterns among different variants. We believe that these efforts will pave the way for precision medicine-based disease management and therapeutic approaches for complex diseases.
